# Exploring Language Impairment in Catalan-Dominant Bilinguals with Primary Progressive Aphasia: Preliminary Data

**DOI:** 10.3390/brainsci15111193

**Published:** 2025-11-04

**Authors:** Io Salmons, Helena Muntané-Sánchez

**Affiliations:** 1 Departament de Filologia Catalana, Universitat Autònoma de Barcelona, 08193 Barcelona, Spain; 2 Hospital de Sant Rafael, 08035 Barcelona, Spain

**Keywords:** primary progressive aphasia, assessment, Catalan, bilingualism, Comprehension Aphasia Test

## Abstract

Background: Research on primary progressive aphasia (PPA) in minority languages and bilingual speakers remains limited, which can compromise accurate diagnosis and intervention. This is the case for Catalan, which lacks standardized tools for this population. Objectives: This study aimed to evaluate the Catalan version of the *Comprehensive Aphasia Test* (CAT-CAT) for detecting and characterizing PPA in Catalan-dominant bilingual individuals. Methods: We administered the CAT-CAT to four participants clinically diagnosed with PPA. Results: The test detected participants’ core linguistic impairments, such as anomia, and revealed distinct severity profiles consistent with PPA variant types. Moreover, it captured deficits that were not identified by routine clinical observation or informal assessment. Conclusions: This study provides the first detailed characterization of PPA in Catalan-speaking individuals and, although based on a small sample, its findings address a critical gap in neurodegenerative language research and highlight the importance of standardized tools to improve diagnosis and guide clinical interventions in bilingual speakers of minority languages.

## 1. Introduction

Since the seminal work by Mesulam [1], who described primary progressive aphasia (PPA) as a neurodegenerative syndrome with progressive language impairment, extensive research has been carried out in the field (e.g., [2,3,4]). Since then, Gorno-Tempini and colleagues [5] established the International Consensus Criteria for diagnosing and classifying PPA into three main variants: the semantic variant (svPPA), the logopenic variant (lvPPA), and the nonfluent/agrammatic variant (nfvPPA). The svPPA is characterized primarily by severe anomia, deficits in semantic knowledge and comprehension, and surface dysgraphia and dyslexia. In contrast, lvPPA involves milder lexical retrieval impairments and deficits in sentence repetition. Finally, nfvPPA is characterized by effortful speech, which may be agrammatic and/or apraxic, along with syntactic comprehension difficulties. However, some patients exhibit mixed symptoms that do not fit into any of the three previously mentioned variants, as noted by Gorno-Tempini et al. [5].

However, most studies have focused on English-speaking individuals and monolingual speakers. Only more recently, researchers have worked on the presentation, assessment, and treatment of PPA in other languages (among others, [6,7,8]) and in bilingual speakers (see [9] and references therein). Despite these efforts, research on minoritized languages remains limited. This is the case for Catalan (see [10] for discussion of its minoritized status), which lacks studies characterizing PPA in Catalan speakers, as well as adapted assessment tools and treatment for this population.

So far, the few studies involving Catalan speakers with PPA have approached them within a bilingual framework, focusing mainly on the comparison of Catalan with the majority language, primarily Spanish [11,12,13], or treating Catalan and Spanish speakers as a single undifferentiated group [14]. While previous studies provide valuable insights, there remains a need for approaches that specifically address the unique assessment and treatment challenges faced by Catalan speakers. Additionally, previous studies focus only on one ability, such as naming or speech. As a result, the language deficits of people with PPA (pwPPA) have not yet been described in Catalan.

This work aims to contribute to addressing that gap, focusing on the assessment and diagnosis of PPA in Catalan-speaking individuals. Standardized tools are crucial for characterizing patients’ language deficits, understanding the nature of their impairments, and designing tailored interventions. To address the lack of standardized assessment tools for Catalan-speaking pwPPA, we propose using the *Comprehensive Aphasia Test* in Catalan (CAT-CAT) [15,16]. The CAT-CAT is the only available tool with published normative data for Catalan to date [17] and has demonstrated sensitivity to subtle language deficits in post-stroke aphasia [18,19]. It is a comprehensive test that evaluates a wide range of language abilities across modalities, including those necessary to diagnose PPA, such as naming, comprehension, production, repetition, reading, and writing [20]. Previous studies have found significant overlap between PPA and post-stroke aphasia (e.g., [21]); thus, despite not being specifically designed for PPA, the CAT-CAT may represent a valuable, practical tool for standardized assessment and monitoring of language deficits in Catalan-speaking pwPPA.

The CAT also has the advantage of having been adapted to multiple languages [22], such as Turkish, Basque or Arabic [23,24,25], and studies have shown that the different versions are comparable (e.g., [26]), facilitating more tailored assessment of bilingual speakers. Moreover, it was developed to assess and monitor language abilities over time [27], making it a potentially suitable tool for tracking the progressive changes characteristic of this syndrome. Based on these features, we expect that the CAT-CAT will detect linguistic deficits in pwPPA. Moreover, we expect Catalan-speaking pwPPA to present with more severe and less specific deficits, likely due to the lack of standardized tests, which often results in delayed diagnoses.

Therefore, this study evaluates the utility of the CAT-CAT for assessing PPA in Catalan-dominant bilinguals, a population often overlooked in the development of standardized assessments. We administered the CAT-CAT to four participants clinically diagnosed with PPA through informal Spanish-language assessments and general cognitive screening tools. Our aims were to: (i) determine the test’s sensitivity to PPA-linguistic deficits (e.g., anomia) in order to evaluate its potential clinical utility as a diagnostic tool; (ii) describe detailed linguistic profiles, sometimes overlooked in non-standardized examinations, that are crucial for accurate variant identification and intervention planning; (iii) conduct a detailed micro-quantitative and qualitative analysis of responses—particularly error types and speech—to characterize the language impairments exhibited by bilingual participants, given that results cannot be validated due to the absence of standardized assessment tools in Catalan; and (iv) provide, for the first time, a description of language deficits in Catalan-speaking pwPPA, addressing a significant gap in the literature.

## 2. Materials and Methods

In this section, we describe the participants, materials, and procedures, and present the research questions derived from the study goals outlined in the Introduction:

1. How sensitive is the CAT-CAT in detecting linguistic deficits in Catalan-dominant pwPPA?
2. What are the detailed linguistic profiles of participants, and how can they inform accurate variant identification and the severity of their language deficits?
3. How do micro-quantitative and qualitative analyses of responses characterize language impairments in bilingual participants?
4. What is the nature of language deficits in Catalan-dominant bilinguals with PPA?

### 2.1. Participants

We examined the language abilities of four participants with primary progressive aphasia (pwPPA), with a mean age of 79.5 years, all right-handed, including one woman and three men, with different educational backgrounds. Demographic and clinical characteristics of participants as reported in medical histories are in Table 1. The participants were recruited from the patient pool of Hospital de Sant Rafael in Barcelona (Spain). They had been diagnosed with PPA by neurologists and referred from neurology units or general practitioners in the surrounding area for speech-language pathology therapy. All pwPPA were early, simultaneous bilinguals and proficient speakers of Catalan and Spanish, with Catalan as their dominant language. All received formal schooling in Spanish and were literate in that language, but they had not been formally taught to write in Catalan.

The clinical diagnosis was mainly based on informal observation of language skills and, sometimes, on informal language tasks administered by neuropsychologists. Moreover, patients were given brief screening tests to evaluate their general cognitive state, with the *Mini-Mental State Examination* (MMSE) [28,29], along with the *Global Deterioration Scale* (GDS) [30] or the *Test de Alteración de Memoria* (TaM; Memory Alteration Test) [31]. None of the aforementioned tests have standardized versions in Catalan. Patients also underwent neuroanatomical examination through different imaging techniques to confirm the diagnosis and, in some cases, histopathological studies to determine the underlying pathology.

Participants were included in the study only when their medical records provided information consistent with the diagnostic criteria proposed by Gorno-Tempini and colleagues [5], while participants with a documented history of stroke, traumatic brain injury, or comorbid neurological or psychiatric conditions were excluded. None of the subjects that participated in the present study had any prior history of neurological or cognitive disorders before the onset of PPA.

In the following paragraphs, we describe the clinical and demographic profiles of each participant in detail. In all cases, language impairment was the primary and predominant symptom at disease onset, while their general cognitive skills were only mildly affected (see Table 1). During the early stages, all the participants carried out most of their daily activities independently and their only complaint was language difficulties, primarily anomia. Medical reports also indicated a progressive deterioration of language over time. The described neuroanatomical events causing the syndrome are heterogeneous, which is common in the PPA population [5]. Despite the predominance of language difficulties in all cases, none of the participants had undergone a formal and comprehensive language assessment.

#### 2.1.1. PPA1

The participant first noticed symptoms one year before consulting a neurologist, primarily involving difficulties with language. Magnetic Resonance Imaging (MRI) showed a lesion suggestive of encephalomalacia in the inferior left temporal lobe; however, no history of brain injury or other neurological events was documented. The attending physician also reported small white matter demyelinating foci in corticosubcortical areas in both hemispheres, particularly in periventricular regions, consistent with vascular changes related to aging and degenerative processes (Fazekas grade 1). He was then diagnosed with an unspecified variant of PPA associated with Alzheimer’s disease. According to reports from the speech-language pathologist and family members, his language abilities declined notably in the months following our assessment.

#### 2.1.2. PPA2

The participant consulted a doctor four years before being diagnosed with PPA, with complaints of word-finding and speech impoverishment. His general cognitive abilities were screened using the TaM and GDS scales, which indicated mild cognitive deterioration. His symptoms were initially attributed to attentional-type errors, due to stress-related problems, given that he was the main caregiver of a stroke survivor. Three years later, he again consulted a neurologist after his daughter expressed concerns about his significant language difficulties. Computed Tomography (CT) revealed white matter hypodensities in both hemispheres of microangiopathic sequelae and signs of cerebral involution, which were confirmed by MRI, especially in the left inferior frontal lobe. A subsequent PET study also showed intense and extensive hypometabolism in the left frontal and parietotemporal lobes. He then received a diagnosis of nonfluent PPA with suspected tauopathy. According to the neurologist’s report, the patient was practically mute five months after our assessment and tended to isolate himself due to his language problems, apparent lack of attention and apparent passive behavior, despite having intact auditory abilities.

#### 2.1.3. PPA3

The participant reported starting to experience problems remembering words years before the first consultation. Her daughter confirmed that she had significant word-finding difficulties, often used circumlocutions, and that her speech tended to be incoherent. According to the physician, the MRI showed atrophy mainly in the left hemisphere, whereas the PET study revealed amyloid deposition in the cerebral cortex. On the other hand, cerebrospinal fluid analysis showed decreased Aβ42, elevated phosphorylated tau, and increased total tau—a profile the neurologist considered consistent with Alzheimer’s disease presenting as PPA. One year after our assessment, the attending neuropsychologist noted that while her nonlinguistic cognitive abilities, such as memory, were stable, her language skills worsened.

#### 2.1.4. PPA4

The participant and his wife consulted with a neurologist after months of experiencing language difficulties, particularly word-finding issues and comprehension problems. As documented in the medical history, his screening results on the MMSE and GDS indicated mild cognitive impairment, although the psychoneurologist concluded that the results were likely influenced by his language difficulties, which were limited to anomia. No significant memory or orientation problems were reported. A CT scan revealed cortical atrophy in the parietal lobes and very mild leukoaraiosis. He was diagnosed with the logopenic variant of PPA. According to the neuropsychological reports from nine months after our assessment, his language abilities had significantly declined, and his profile was by then more consistent with the nonfluent variant of PPA.

### 2.2. Assessment

The participants were administered the Catalan version of the CAT [15,16]. The CAT-CAT includes twenty-seven subtests that evaluate relevant domains of language for PPA assessment, as outlined by Nickels and Croot [32], specifically production, repetition, naming, comprehension, reading and writing. In addition, the test contains a short battery of nonlinguistic cognitive abilities to evaluate the presence of deficits such as apraxia, agnosia or acalculia. It is thus a comprehensive test that provides a general profile of the linguistic and nonlinguistic abilities of individuals, has already been used with other populations with language deficits, and is the only currently available tool standardized for Catalan-speaking individuals. The complete list of subtests is provided in Table 2, whereas the materials for the adaptation into Catalan are available at https://ddd.uab.cat/record/250143 (accessed on 12 June 2024).

### 2.3. Procedure

Participants were assessed with the CAT-CAT prior to receiving speech-language pathology therapy. The test was administered in two sessions, each lasting approximately 1 h, by a speech-language pathologist in a quiet, distraction-free environment. Each task was explained to the participant, followed by a practice item to ensure they understood the instructions. Feedback was provided only during the practice items. The administrator manually transcribed all responses, except for the oral descriptions, which were recorded and later transcribed, reviewed, and scored by two researchers. The annotated transcriptions analyzed in this study are available in the Supplementary Material (see also Appendix B.1 for sample excerpts).

Several tasks (subtests 7–18 and 20–23) included two scoring systems: an accuracy-based system, in which correct answers score 1 and incorrect answers score 0; and a second one that uses a 0–2 scale, in which incorrect responses score 0, correct prompt responses score 2, and correct responses after a five-second delay, upon repetition of the item on request, or through self-correction, score 1. Additionally, all participants responded in Spanish in some subtests. These responses were considered errors in the oral subtests. This criterion was based on findings in age-matched controls from the normative study [17], who produced very few words in Spanish in these tasks (e.g., the greatest number of responses in Spanish was observed in the verbal fluency subtests, where 2.3% of the responses were in Spanish). In written tasks (subtests 25–27), Spanish responses were accepted and included in the analysis, reflecting that participants over the age of 70 often responded in Spanish in the normative sample. This behavior demonstrates their lack of formal education in Catalan, which was forbidden during the fascist dictatorship in Spain (1939–1975), rather than a deficiency in their language skills. Accordingly, for one participant who explicitly stated an unwillingness to write in Catalan, subtests 25 and 26 were administered using the Spanish version of the CAT [33].

### 2.4. Data Analysis

The data analysis and visualization were conducted using R software (RStudio version 2025.05.0+496) [34]. Given the small sample size and the exploratory nature of this study, only descriptive statistics were performed. Inferential analyses were not conducted because they would not provide reliable results in this context. Therefore, descriptive statistics are reported, particularly the maximum possible score and the participants’ raw scores for each subtest. In addition, the severity of impairment was calculated for each subtest and participant based on the difference between the participant’s score and the cutoff, and was normalized by the range between the cutoff and the maximum scores to allow comparison across subtests. Cutoff scores were based on a separate normative study with 110 healthy participants [17]. The article describing this study is still under revision, but the dataset is fully available for verification [35].

In addition, we conducted an acoustic analysis of the oral descriptions to complement the fluency and content ratings, given that the CAT-CAT is not standardized for PPA populations. Recordings of oral descriptions were manually segmented into speech and pauses using the Praat software [36]. Pauses were only considered if they lasted longer than 250 milliseconds [37], and were classified into silent pauses and filled pauses. Pauses occurring immediately before and after the intervention of the speech-language pathologist were excluded to avoid including pauses influenced by the intervention rather than the participant’s speech.

## 3. Results

### 3.1. Overall Results and Severity Profiles

The performance of the four participants on the CAT-CAT varied across tasks, but all obtained scores below the cutoff thresholds in several subtests (highlighted in Table 2 in bold), indicating impaired performance across various language domains. The results of the language battery showed that all participants had deficits in naming objects, as well as in comprehending spoken words and sentences, and in writing to dictation. In addition, their form scores on written descriptions were below the threshold. As for their results on the cognitive screen, all participants performed below the normal threshold on the semantic word fluency subtest, which also evaluates verbal skills, and on the arithmetic subtest, which assesses acalculia.

**Figure 1:**
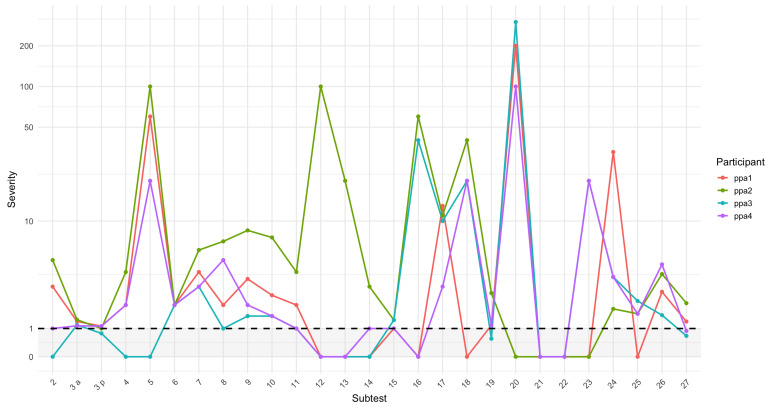
Severity scores by subtest: 0 = maximum; 1 = cutoff; and above 1 = impairment. Subtest 3a = animals; 3p = words starting with p. The dashed line indicates the cutoff value.

The deficits observed across language domains, however, varied in severity. Since the different subtests also differ in difficulty, we calculated the severity index scores for each participant and subtest (Figure 1; see Appendix A.1 for the raw scores). Scores from 0 to 1 reflect unimpaired performance: a score of 0 indicates that the participant obtained the maximum score, whereas a score of 1 corresponds to performance at the cutoff level. Scores above 1 indicate increasing levels of impairment. Results from subtests 5, 12, 13, 16, 18, 21, 22, and 23 should be interpreted with caution and with respect to raw scores, due to ceiling effects in the normative sample that in some cases inflated severity values.

With these considerations in mind, we examined the distinct severity profiles for each participant. For instance, participant PPA2 exhibited the most severe impairments, with difficulties across all linguistic domains except for reading subtests (20–23), where he performed at ceiling. His most severe deficits were in comprehension (subtests 7–11) and production (subtests 19 and 27). Cognitive screening also revealed deficits in semantic memory tasks (subtests 2 and 4), motor abilities (5) and calculation skills (6). In contrast, PPA3 presented with more specific and less severe deficits, primarily in naming (subtests 3, 17, 18, and 25) and spelling (25–26), as well as in working memory (15–16).

PPA4, on the other hand, exhibited a similar profile but with spared working memory, although his digit span score fell at the lowest end of the normal range, and he showed slightly worse performance on visual recognition memory and gesture object use. Lastly, PPA1 achieved the lowest scoring on the oral object naming subtest among the four participants. His performance also revealed problems in written production and spelling (26–27), as well as in nonlinguistic cognitive abilities, such as those assessed by the semantic subtests (2 and 4). His score on the copying task was very low, though this was due to his failure to follow instructions to copy the words in capital letters throughout the test (see an example in Figure A2 in Appendix B.2).

### 3.2. General Cognition

The CAT screens for cognitive deficits (subtests 1–6 and 15–16), which can provide information about factors that may influence language performance, as well as the course and symptom profile of the disease. As already mentioned, all the participants performed below the cutoff in the arithmetic and verbal fluency tasks, though the latter will be examined along with the naming subtests. Additionally, three out of the four participants exhibited difficulties on the gesture object use subtest, which screens for limb apraxia.

Furthermore, two participants scored below the threshold in the subtest that assesses semantic knowledge; notably, all errors—regardless of overall score—involved the selection of depictions of semantically related objects. The three participants who made errors on the semantic memory task performed below the cutoff on the recognition memory subtest, which required recalling the same items presented in the former task. This subtest allows for assessment of visual short-term recognition memory, whereas subtests 15 and 16 assess verbal short-term memory. In the digit span subtest, two participants performed above the cutoff, although at the lower end of the normal range, and two fell below the threshold. These two participants also scored below the normal range in the word span task, indicating working memory deficits, while the other two performed at ceiling.

### 3.3. Comprehension

**Figure A1:**
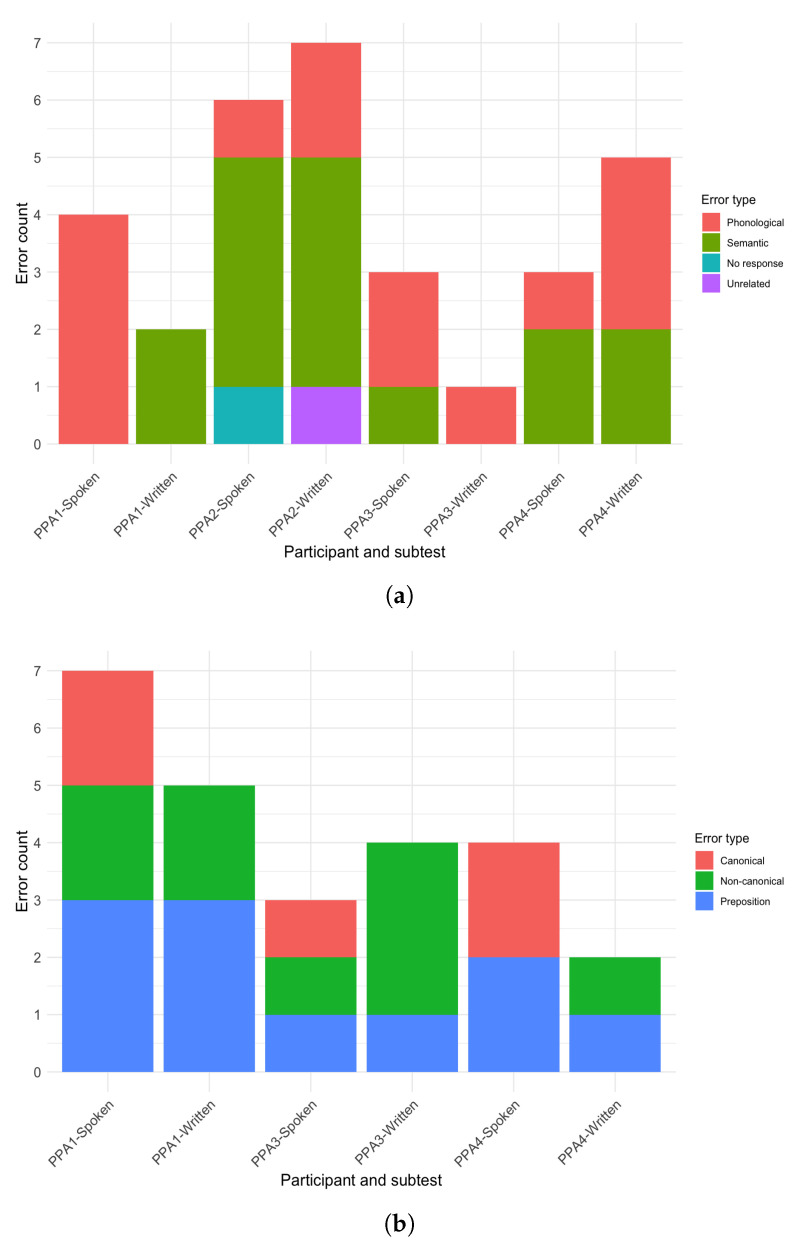
Error types per participant in comprehension subtests. (**a**) Word comprehension (subtests 7 and 8). (**b**) Sentence comprehension (subtests 9 and 10).

**Figure A2:**
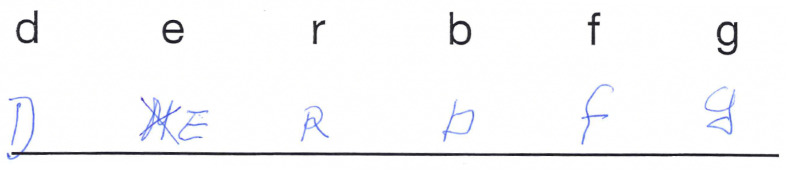
Sample response from PPA1 in the copying subtest.

Comprehension was the domain in which all participants made errors (see Figure A1a in Appendix A.2). PPA2 made the greatest number of errors across comprehension subtests, including near-floor performance on the sentence comprehension task, indicating a severe deficit. The other three participants’ errors in the word comprehension subtest involved both phonological and semantic distractors. With respect to sentence comprehension, their errors involved semantically reversible sentences (Figure A1b): canonical sentences with two arguments or with prepositions, and non-canonical structures, such as passives and dislocations. Most errors (21 out of 24) consisted of the selection of the image depicting role reversal (e.g., PPA1 pointed to the image showing a glass placed on top of a plate when he heard the sentence *El got és sota el plat*. ‘The glass is under the dish.’). Of these three participants, PPA1 shows the most severe deficit in sentence comprehension.

### 3.4. Word Production

The oral production of words was assessed across modalities, particularly naming and repetition. All participants except PPA2 performed at ceiling or near-ceiling levels on repetition subtests. For instance, only one participant, PPA4, made a single error, producing the word *gust* ‘taste’ instead of the nonword *guts*. PPA2, on the other hand, made errors across all word types. In most cases, he produced phonologically similar words, for example, he substituted the derivational morpheme in *actualitat* ‘current events’ with another morpheme *autualitzar* (*actualitzar*, ‘to update’).

All pwPPA scored below the cutoff threshold in naming subtests, with most errors consisting of no responses (22 out of 40 errors; see Table A2 in Appendix A.2) and at least one response in Spanish. PPA1 produced a majority of semantic errors, such as circumlocutions or semantic paraphasias. In contrast, PPA2 was the only participant who also produced perseverations. PPA3 did not respond initially, but when asked, she was able to describe the images using circumlocutions; for example, she used the expression “a house for working” for *factory*. Finally, PPA4 presented the least severe deficit, and the few errors he made involved both semantic and phonetic paraphasias. Notably, all participants also made more mistakes with low-frequency nouns than with high-frequency ones (30 versus 7 errors, respectively). Similarly, they also tended to have more difficulty naming words with three or four syllables (22 errors) than shorter ones (15 errors), although the difference was less pronounced.

In the verbal fluency subtests, most pwPPA showed better performance on the semantic criterion than on the phonological one (Table 2). Besides the number of appropriate nouns in Catalan that they produced per minute, the number and type of errors were also taken into account (see also Table A2). PPA2, who produced the fewest words, made no mistakes, while PPA1 repeated one correct item in the semantic task. PPA4 made various errors, including circumlocutions in the semantic criterion and category errors (animals) in the phonological task. PPA3 produced inappropriate words only in the phonological task, namely two words in Spanish and one proper name.

### 3.5. Reading and Spelling

The CAT includes several tasks to assess reading skills across different word types. Additionally, spelling abilities are evaluated through various modalities, including letter and word copying, naming, writing to dictation, and describing an image. Importantly, all participants responded at least partially in one or more written subtests in Spanish (see Appendix B.2 for examples) and one of them, PPA4, completed subtests 25 and 26 from the Spanish version of the CAT [33], as per his request. Catalan orthography is more transparent than English but less so than Spanish, with frequent correspondence between spelling and sound. For this reason, the Catalan and Spanish versions of the CAT do not categorize items in terms of regularity [17,33], especially in reading subtests, which are consequently less sensitive to certain deficits. Catalan partially does so for writing subtests, though irregular words involve semi-transparent orthography in some dialects, as illustrated by errors like *restauran* for *restaurant* below. Nevertheless, as previous studies have argued [38], a careful analysis of error types can reveal patterns of response that are informative about the nature of the deficit and relevant for diagnostic purposes of certain PPA variant types.

PPA1 correctly read and wrote pseudowords throughout the tasks. He produced very few reading errors; for instance, the only two mistakes involved similar words (e.g., *Eixample* (a neighborhood in Barcelona) instead of *exemple* ‘example’). However, he experienced more difficulties with spelling, including phonological errors, such as omitting letters that are not pronounced (e.g., *restauran* instead of *restaurant* in the copying subtest). In addition, he also had problems writing consonant clusters such as *bl/br* and the letter *ñ* in Spanish. Interestingly, he responded in Spanish only in the dictation and written description subtests.

**Figure A3:**
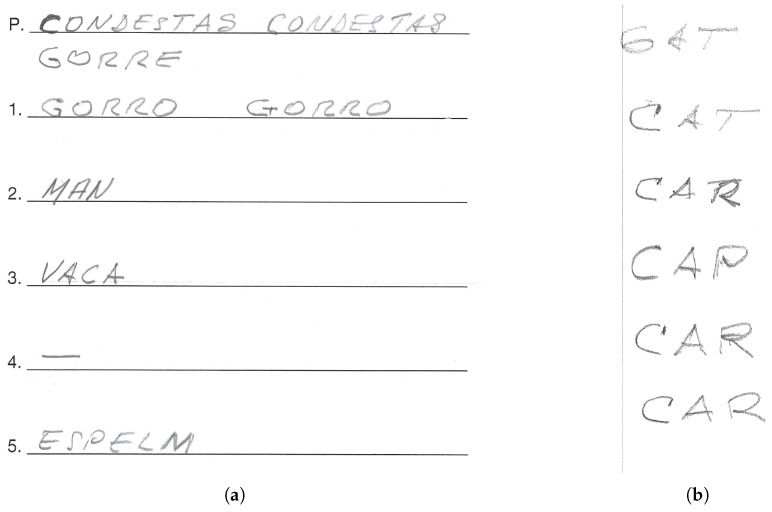
Written responses from PPA2. (**a**) Responses from the written naming subtest: *P. CONDESTAS CONDESTAS GORRE, 1. GORRO GORRO, 2. MAN, 3. VACA, 5. ESPELM*; translation: ‘[neologism], [neologism], cap (Spanish), cap (Spanish), hand, cow, candle’; target responses and translation: *barret* ‘cap’, *nen* ‘child’, *mà* ‘hand’, *vaca* ‘cow’, *iglú*, ‘igloo’, *espelma* ‘candle’. (**b**) Description of the image in subtest 27: *GAT, CAT, CAR, CAP, CAR, CAR*; translation: ‘cat, [neologism], [neologism], head, [neologism], [neologism]’.

In contrast, PPA2 showed significantly more severe deficits in production, as evidenced by automatisms, such as verbal perseveration, and anomia (see Figure A3 in Appendix B.2). While his reading abilities were spared, his writing errors in the naming and dictation subtests included phonologically similar words and nonwords (e.g., *foc* ‘fire’ instead of *pot* ‘jar’ and *man* instead of *mà* ‘hand’). Interestingly, in the dictation subtest, he was unable to spell the pseudoword and made phonologically based errors with irregular words written as pronounced (e.g., *impansable* instead of *impensable*).

PPA3 and PPA4 showed a similar pattern of spelling errors, all of which shared phonological similarity with the target. The errors involved all types of words: pseudowords (e.g., *pur* instead of *plurc*), irregular words (e.g., *ada* instead of *hada* (Spanish) ‘fairy’), regular words (e.g., *abica* instead of *abeja* (Spanish) ‘bee’), and even complex words (*iomoda* for *incomodidad* (Spanish) ‘discomfort’). Regarding the reading tasks, the few errors that these two participants made were also phonologically related to the target.

### 3.6. Description Tasks

Speech analysis is central for assessment and diagnosis of PPA, as discussed in Ossewaard et al. [39] and references therein. The CAT-CAT includes a closed-class scoring system that allows for content and form variables to be evaluated separately (see Appendix A.3 for raw scores and further details). As in other subtests, PPA2 was the participant with the lowest scores, all of which fell below the cutoff. The other participants showed more selective impairments (Table A3). With regard to content variables in the oral description, they all exhibited low informativeness, producing only 0% to 66.7% of the relevant content units, but PPA1 produced a lower proportion of relevant nouns than verbs, while PPA3 showed the opposite pattern. Interestingly, productivity did not correspond to discourse efficiency, as the number of nouns and verbs produced did not translate into a greater number of relevant nouns and verbs (see Table A4); for example, PPA4 produced 20 verbs, of which only 2 were relevant, whereas PPA1 produced 6 nouns, 4 of which were relevant.

In addition, the error analysis revealed distinct profiles across participants (see samples in Appendix B.1). PPA1 exhibited predominantly anomic errors, with frequent use of filler words and circumlocutions. For instance, the most produced word by this participant was the non-specific deictic expression *aquí* ‘here’ (11 occurrences). By contrast, PPA2 produced more effortful, shorter and less complex sentences. PPA3, on the other hand, demonstrated greater verbosity, and although she was the most fluent, her description was less coherent and sometimes difficult to follow. She was also the participant that switched to Spanish most frequently. Finally, PPA4 exhibited anomia as well, but his errors were mainly phonetic paraphasias.

**Figure 2:**
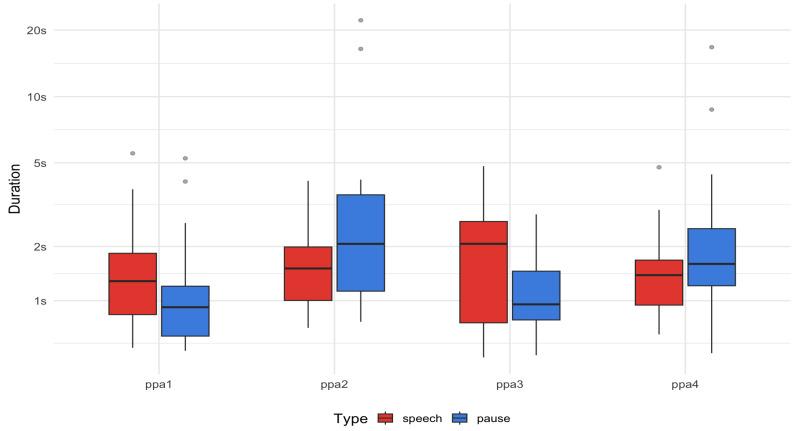
Speech and pause durations (silent and filled) in seconds per participant. Boxplots show the median (line), 25th–75th percentiles (box), and range (whiskers) of durations per participant.

As for form variables, they all scored below the cutoff on fluency, with the exception of PPA3. In fact, we measured the duration of both speech and pauses (silent and filled) to assess whether these quantitative measures (Figure 2; see also Table A5) aligned with the CAT-CAT scoring outcomes (Table 2). The results showed alignment: for example, PPA1 and PPA3 both produced longer speech (means of 1.52 s and 2.03 s, respectively) than pause durations (means of 1.22 s and 1.03 s, respectively). However, this contrast was less pronounced in PPA1, whose shorter speech segments reflected more frequent alternation between brief utterances (37) and short pauses (22), hence, less fluency. In contrast, PPA2 and PPA4 produced longer pauses (means of 6.2 s and 2.58 s, respectively) than speech durations (means of 1.79 s and 1.51 s, respectively). In particular, PPA2’s pauses were very long and frequent (12 utterances, 8 pauses), which significantly affected his level of fluency. PPA4’s pauses were more consistent and slightly exceeded speech durations, indicating a more balanced speaking and pausing pattern (36 utterances, 29 pauses), but still reduced fluency.

**Figure A4:**
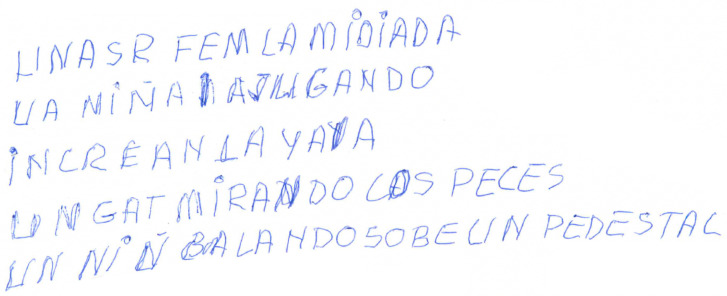
Response from PPA4 in the written description subtest: *UNA SR FEM LA MIDIADA/UA NIÑA AJUGANDO/INCREAN LA YAYA/UN GAT MIRANDO LOS PECES/UN NIÑ BALANDO SOBE UN PEDESTAL*; translation: ‘A lady taking a nap. A girl playing. (They) scold the grandma. A cat watching the fish. A child dancing on a pedestal.’

The four participants performed worse on the written description task than on the oral one, in both content and form variables, with a greater decline observed in the latter (Table A3), mainly due to less complex structures. PPA2 was also the participant who showed more severe impairments, as his written production included repeated content suggestive of perseveration (Figure A3b in Appendix B.2). The other participants produced a low number of relevant content units, although they showed different patterns of impairment: PPA3 and PPA4 named a greater number of relevant objects and actions than PPA1. Interestingly, all participants responded in Spanish, except for PPA4, who wrote the first sentence in Catalan (Figure A4). Since the written descriptions were considerably shorter than the oral ones, the participants made fewer mistakes. Most of them involved word spelling errors, such as letter omissions and substitutions, similar to the ones previously described.

## 4. Discussion

The four participants studied were diagnosed with PPA based on clinical and neuroimaging criteria, and two of them, PPA2 and PPA4, were classified as nonfluent and logopenic variants, respectively. However, their language abilities had only been assessed informally or through general cognition screening tools, and primarily in their second language. We evaluated their linguistic abilities with the *Comprehensive Aphasia Test* in Catalan, their dominant language, to assess whether it could detect specific linguistic deficits and reveal distinct impairment profiles, despite not yet being specifically standardized for PPA. This section discusses the results in relation to the potential use of the CAT-CAT, and considers the study’s limitations and directions for future research.

### 4.1. Common Patterns

As expected, the results showed considerable intersubject variability among the participants, reflecting the characteristic heterogeneity of the syndrome [4,40], and particularly with respect to disease stage at assessment, as well as anatomical and etiological differences within the sample. However, they all shared certain features. For instance, the fact that all participants showed deficits in object naming and verbal fluency is consistent with the clinicians’ descriptions in their medical reports and with the literature, according to which anomia is the core deficit in PPA, present across all variants and among the earliest symptoms [32]. Moreover, they performed poorly on the subtests assessing spelling, as documented across all variants in previous literature [41,42], as well as in word and sentence comprehension, with varying levels of severity. Notably, errors occurred across both canonical and non-canonical structures, a pattern that rules out the possibility of agrammatism as the primary source of the deficit. Finally, all participants exhibited impairments in oral descriptions, which will be examined individually in the following section.

One of the advantages of the CAT is that it includes a nonlinguistic cognitive screen, which revealed that the participants had impairments in domains beyond language. Given the progressive nature of the syndrome, examining non-linguistic cognitive abilities is particularly relevant in pwPPA, especially since our participants were assessed at a mean of 38.5 months post-onset. In fact, all participants showed deficits in calculation, and three exhibited impairments in praxis. Calculation deficits have been reported in pwPPA and Alzheimer’s disease, especially when there is involvement of the inferior parietal lobe [43], as is the case for participants PPA2 and PPA4, and suspected in PPA1 and PPA3 based on clinical and biomarker findings. Similarly, apraxia can co-occur with PPA and does not exclude its diagnosis [5]. In any case, the CAT includes a subtest that screens for limb praxis, which can interfere with gesture-based rather than oral communication. Despite these shared features, individual profiles revealed marked differences across domains and in degrees of severity.

### 4.2. Individual Profiles and Variant Type

Given the small sample and the aim to evaluate the clinical utility of the CAT-CAT for diagnosis and guiding intervention planning, we analyze the individual participant profiles and discuss their alignment with the three established PPA variants.

#### 4.2.1. PPA1

Participant PPA1, although diagnosed with an unclassified type of PPA, exhibited a pattern compatible with the semantic variant. On one hand, he showed significant deficits both in naming and description subtests, as well as in nonverbal semantic and recognition memory subtests. As a result, his word and sentence comprehension were compromised. His speech also presented characteristics of the semantic variant [44], in particular, the production of a high number of non-specific words, as well as circumlocutions, which affected the informativeness of the discourse despite being the participant who produced the highest number of words (Appendix A.3). By contrast, the form variables showed intact grammatical abilities and no motor-speech impairments were observed.

On the other hand, his other abilities, such as repetition, were relatively spared. Despite the transparency of Catalan and Spanish orthographies and, hence, the limitation this imposes in the diagnosis of dysgraphia [45], his errors in the subtests assessing spelling revealed patterns that could be consistent with surface dysgraphia [41]: his errors with irregular words were phonologically plausible, while he accurately wrote pseudowords. Yet, he also exhibited additional orthographic impairments with certain letters. Regarding his unimpaired reading skills, which rule out a surface dyslexia diagnosis, the results align with those reported in the literature for Spanish-speaking populations with svPPA [46,47]. These deficits, together with the neuroimaging findings of lesions in the left inferior temporal lobe and white matter changes in periventricular regions, support the diagnosis of semantic variant PPA.

#### 4.2.2. PPA2

Participant PPA2 was diagnosed with nfvPPA on the basis of his speech difficulties, which were mostly described as disfluent and severely anomic. For instance, the participant performed poorly on naming subtests, and his speech was filled with frequent and long pauses that significantly affected its fluency. However, the CAT-CAT also revealed severe linguistic deficits across several domains, which are compatible with the extensive brain damage in language-related regions, especially the left inferior frontal and parietotemporal lobes, reported in his medical records. In particular, his comprehension and production abilities were severely compromised, and he also exhibited varying degrees of deficits across nonlinguistic and linguistic tasks, with the sole exception of reading and copying, where his performance was at ceiling. Notably, he presented difficulties that are more common in other variants; for example, his comprehension problems were not restricted to more complex syntactic structures, he made several semantic errors in the word comprehension subtests, and he presented semantic memory deficits.

These impairments suggest that, at the time of assessment, the participant presented a more severe and mixed type of PPA rather than the nonfluent variant. The fact that he was assessed more than four years after the onset of the first symptoms could explain the progression and broadening of his deficits. This is further supported by the clinician’s observation, months later, that the syndrome had progressed to mutism and that some behavioral signs had started to appear. However, the passive behavior and attention difficulties described in the records may be due to his overlooked language impairments, particularly in the comprehension domain. In fact, if attentional issues had been the primary cause of these difficulties, we would have expected more generalized performance issues throughout the tasks, with more inconsistent errors and difficulties in finishing the test. Yet, his errors, such as semantic and phonetic paraphasias, were more indicative of language processing deficits.

#### 4.2.3. PPA3

Participant PPA3 was diagnosed with an unclassified form of PPA secondary to Alzheimer’s disease and presents with the mildest deficits among the participants. The pattern of impairment observed in the CAT-CAT is compatible with the logopenic variant, as she exhibits the core features, namely anomia and phonological short-memory deficits, which may in turn contribute to her relatively mild comprehension difficulties. Her phonologically based spelling impairments across all types of words are also compatible with this variant [41]. In addition, her general cognitive abilities and certain linguistics skills are relatively spared, such as semantic memory or repetition. Moreover, neuroimaging findings of left hemisphere atrophy, along with the confirmed Alzheimer’s disease etiology, further support this classification [48].

Finally, her oral description also showed some features that could be compatible with lvPPA, as described in previous literature [49]; in particular, despite being fluent, she exhibited frequent false starts and filled pauses, with filled pauses accumulating a notable duration relative to silent pauses (Table A5). In addition, she displayed greater verbosity and difficulty maintaining focus. For example, she finished her description with the sentence *Bueno, les flors aquestes. No sé t-t-t… (1.45, filled) Per això s’ha sortit a fora.* (‘Well, these flowers. I don’t kno-kn-know… That’s why he/she/it went outside.’). Notably, no figure in the image is actually going outside. Moreover, she made some phonological errors, sometimes resulting in unintelligible sequences, while her grammatical abilities were spared.

#### 4.2.4. PPA4

Participant PPA4 was diagnosed with lvPPA based on observed atrophy in the parietal lobes and naming deficits; however, his results on the CAT-CAT only partially fit the variant. He exhibited anomia, milder than that of the other participants in the naming subtests, but more obvious in the description task, where the pause placement in his speech suggested that his disfluency was related to lexical retrieval problems. These results are consistent with previous findings indicating that lvPPA can present with both fluent and non-fluent speech [50]. Unlike PPA3, his oral description was more focused and less verbose, but he exhibited other characteristics consistent with lvPPA [51], namely the aforementioned frequent pauses reflecting naming deficits and the fact that he expressed difficulty in carrying out the task several times (e.g., *És massa liàs.* ‘It’s too confusing.’). However, he made both phonological and semantic errors across different subtests, the latter being more common in svPPA.

In addition, he showed a broad pattern of phonological errors in spelling subtests and comprehension deficits similar to those described for PPA3, as expected in lvPPA. However, his performance on verbal short-term memory was above the cutoff threshold, despite his digit span being at the lower end and his visual recognition memory falling below the cutoff. Short-term memory deficits are one of the classification criteria for lvPPA and are often argued to underlie its comprehension deficits. In addition, he showed mild deficits in semantic memory, also typical of svPPA. The profile at the time of assessment may therefore represent a case with features that do not clearly align with the established classifications. This ambiguity could be attributed to a more advanced stage of the disease, which often manifests as overlapping variants [52].

### 4.3. Clinical Utility of the CAT-CAT and Limitations

The main goal of this study was to explore whether the CAT-CAT could be used to assess and diagnose pwPPA in Catalan-speaking individuals. The results discussed above show that the test detected impairments across several linguistic and nonlinguistic subtests in all participants. The observed deficits not only aligned with the core features of PPA, such as anomia, but were also sensitive to distinct impairment and severity profiles, some of which potentially compatible with classic variant types, as defined in Gorno-Tempini et al. [5]. More importantly, the results were generally compatible with the participants’ medical records, which described the results of neuroimaging studies, clinical observations, and patients’ complaints. Interestingly, the CAT’s brief general cognition screening test also revealed cognitive deterioration, complementing the detailed language assessment.

Moreover, our assessment showed great sensitivity, as it captured deficits that were overlooked or misattributed in clinical explorations. For example, despite PPA4 reporting difficulties with comprehension during the initial consultation, the medical records consistently attributed these issues to auditory problems rather than true language comprehension deficits. This perspective was maintained throughout follow-up visits before our assessment. However, the CAT-CAT rules out the possibility of purely auditory deficits: on one hand, he performed at ceiling on repetition subtests that rely heavily on auditory processing, and on the other hand, he exhibited clear difficulties in both written word and sentence comprehension. Likewise, the patient showed difficulties in spelling, contrary to the medical records, which stated there were no alterations in writing. It is important to note that these writing impairments cannot be attributed to differences in L1 and L2, as the patient’s spelling was assessed using the Spanish version of the CAT.

Similarly, the language difficulties reported by PPA2 and his relatives were initially attributed to stress-related problems, resulting in a four-year delay in PPA diagnosis. As with the previous patient, the medical records mentioned social isolation and decreased attention, but not comprehension problems, even months after our assessment. However, the results of this study showed that PPA2 presented a severe deficit in comprehension cannot be explained by attentional deficits, as discussed above. The fact that the CAT-CAT detected distinct linguistic impairments, including those overlooked in clinical explorations, suggests that it could be a valuable tool to assess and diagnose Catalan-speaking pwPPA. This further emphasizes the need of comprehensive language assessment for early and accurate diagnosis.

In addition, the variability of symptoms and the difficulty in classifying some of the patients highlight the critical need for detailed linguistic profiling rather than reliance on general cognition screenings or informal tests alone. This is partly due to the heterogeneous nature of the syndrome, but it can be exacerbated by the lack of standardized tools available for minority-language speakers. Our participants’ histories (e.g., PPA2’s four-year diagnostic delay and severity, the presence of general cognitive deficits…) exemplify the impact of current gaps in the assessment of Catalan-speaking individuals. In fact, one novel aspect of this study is its focus on the dominant language of bilingual speakers, whereas previous bilingual research has examined PPA mainly in relation to or focused solely on the majority language (e.g., Spanish in [53]).

### 4.4. Bilingualism in Minority Language Speakers

The results revealed some of the challenges often encountered when examining bilingual speakers of minority languages, for whom language-mixing and code-switching behavior is very common, even in individuals without language disorders. For instance, the normative study with 110 control participants revealed interferences from L2 (Spanish) in the verbal fluency subtests and in the oral description, mostly at the lexical level, but not in other oral tasks [17]. However, these interferences were not significant: for example, in the oral naming subtest, only 5 out of the 2640 responses were in Spanish, and in the naming subtests, 55 out of a total of 2886 responses. Hence, a high proportion of interferences from L2 in oral subtests should be considered non-normative.

In this study, all pwPPA switched to Spanish in written subtests and occasionally responded in Spanish in naming tasks. Based on the normative data, the pwPPA’s responses in Spanish during the naming subtests and PPA2’s interferences in the writing-to-dictation subtest are atypical and could be considered pathological. Although several studies have observed language-switching behavior as a communication strategy in individuals with language disorders [54], the patterns of response observed in the pwPPA suggest otherwise. For example, in the object naming subtest, the most frequent errors were non-responses, which indicates that the strategy of switching to the second language was either not available or not efficient.

However, also taking the normative data into account, responses in Spanish in the written subtests should be interpreted differently to avoid underestimating preserved language abilities. As noted, their code-switching behavior in written tasks is probably due to their lack of formal education in Catalan, and it was also observed in participants without disorders of the same age who tended to oversimplify and shorten their descriptions to avoid writing in Catalan. This suggests that pwPPA could have been switching to their L2 in written description as a communication strategy.

While there are no previous studies on Catalan-dominant bilinguals with PPA, our findings align with Gómez-Ruiz et al. [55] who reported that Catalan-dominant bilinguals with Alzheimer’s disease performed better in their L2 (Spanish) on written subtests. Similarly, the review of studies on multilinguals with PPA and dementia conducted by Malcolm et al. [9] reported that, while L1 was usually better preserved than L2, dominance did not always explain performance. We suggest that dominance interacts with sociocultural factors related to the unbalanced status of minoritized versus majority languages, and that individuals may show dominance in different languages depending on the domain or context. It is important to note that the observed preference for Spanish in written tasks in this study does not indicate that L2 is inherently better preserved, as suggested by previous studies [55], but rather reflects limited opportunities to develop L1 writing skills.

This is consistent with the hypothesis postulated by Green and Abutalebi [56], according to which language control processes in bilinguals adapt to the demands of different contexts. In line with this model, the preference for Spanish in written tasks among the pwPPA may reflect adaptive control rather than a deficit, as participants select the language that maximizes communicative efficiency in a given context. For the pwPPA in this study, Spanish was the most used language for writing, which would explain the interferences from Spanish in the written subtests (e.g., PPA2’s responses in the writing-to-dictation subtest were in Spanish). However, one participant chose to complete the written tests in Spanish because he was aware of his difficulties in Catalan, which was unrelated to cognitive control processes. Further research with a larger sample size is needed to clarify this issue.

These findings further highlight the importance of using standardized tests for the assessment of minority languages to obtain an accurate linguistic profile, as the bilingual status of the participant could affect the interpretation of assessment results. In addition, they also emphasize the need for flexible and tailored assessments in minority-language populations; for example, conducting oral subtests in Catalan and written subtests in Spanish, as in this study. The advantage of the CAT is that its multilingual availability allows flexible administration across languages, such as Catalan and Spanish. This enables clinicians to customize the assessment to the patient’s dominant language in each domain, especially in time-limited clinical settings, thereby maximizing diagnostic accuracy and facilitating the design of more effective interventions.

## 5. Conclusions

This study evaluated the Catalan version of the *Comprehension Aphasia Test* for detecting and characterizing PPA in Catalan-dominant bilingual individuals. Our findings showed that the test was sensitive to the deficits presented by the four participants: it detected core linguistic impairments, such as anomia, and revealed distinct severity profiles consistent with PPA variant types. Moreover, it captured deficits that were not identified by routine clinical observation or informal assessment. Despite its limitations, this study emphasizes the need for language assessment tools to assist in the clinical diagnosis of primary progressive aphasia, whose initial symptoms often go unnoticed otherwise.

As the first study to systematically characterize PPA in Catalan, this work fills a critical gap in neurodegenerative language research and provides a foundation for more equitable clinical practice. While no current treatments prevent PPA progression, early diagnosis enabled by tools like the CAT-CAT facilitates interventions that can improve communication and quality of life for patients and their relatives. Recent studies have shown that interventions can be effective for pwPPA in early stages (e.g., [57]). However, without an accurate assessment, interventions cannot be targeted effectively. For Catalan-speaking PPA patients, the CAT-CAT represents a crucial first step toward equitable care.

Nonetheless, this preliminary study has some limitations, including a small sample size, which future research should address, as our findings are exploratory and cannot be generalized beyond the current sample. This study was intended to assess the feasibility of administering the CAT-CAT in Catalan-speaking PPA patients and to generate initial descriptive data. These preliminary results will inform the design of larger-scale studies aimed at validating the tool’s clinical utility, including its ability to diagnose PPA, distinguish its variants, and evaluate potential therapy outcomes following L1-language interventions.

## Figures and Tables

**Table 1:** Characteristics of participants.

Participant	Age	Sex	Education	Subtype	TPO (Months)	Etiology	Cognition Assessment
PPA1	79	male	higher	unspecified	32	Alzheimer’s disease	NA
PPA2	89	male	intermediate	nonfluent	50	Probable tauopathy	TaM: 38, GDS: 3
PPA3	71	female	basic	unspecified	48	Alzheimer’s disease	MMSE: 24, GDS: 4
PPA4	79	male	higher	logopenic	24	Alzheimer’s disease	MMSE: 23, GDS: 3

NA: no data available; TPO: time post onset, based on patient’s reports as reflected in the medical history.

**Table 2:** Results on the CAT-CAT.

Subtest		Maximum	PPA1	PPA2	PPA3	PPA4
Cognitive screen						
1	Line bisection	±6	−2	−1	N.T.	N.T.
2	Semantic memory	10	**7**	**5**	10	9
3	Word fluency					
	animals	–	**2**	**0**	**6**	**7**
	*p*	–	**0**	1	3	**0**
4	Recognition memory	10	**8**	**6**	10	**8**
5	Gesture object use	12	**9**	**7**	12	**11**
6	Arithmetic	6	**2**	**2**	**2**	**2**
Comprehension						
7	Spoken words	15–30	**11**–**22**	**9**–**17**	**12**–**21**	**12**–**24**
8	Written words	15–30	**13**–**24**	**8**–**15**	14–29	**10**–**18**
9	Spoken sentences	18–36	**11**–**21**	**1**–**1**	**15**–**28**	**14**–**27**
10	Written sentences	18–36	**13**–**24**	**3**–**4**	**15**–**29**	**15**–**27**
11	Paragraphs	4	**2**	**0**	3	3
Oral repetition						
12	Simple words	16–32	16–32	**11**–**21**	16–32	16–32
13	Complex words	3–6	3–6	**2**–**3**	3–6	3–6
14	Nonwords	3–6	3–5	**0**–**0**	3–6	2–4
15	Digit span	7–14	4–8	**3**–**6**	**3**–**6**	4–8
16	Word span	6–12	6–12	**3**–**6**	**4**–**8**	6–12
Oral naming						
17	Nouns	24–48	**11**–**21**	**13**–**26**	**14**–**28**	**21**–**39**
18	Verbs	5–10	5–10	**3**–**6**	**4**–**8**	**4**–**8**
Oral description						
19	Content	19	11	**5**	14	12
	Form	9	**8**	**2**	9	**7**
	Total	28	**19**	**7**	23	**19**
Reading						
20	Simple words	24–48	**22**–**44**	24–48	**21**–**41**	**23**–**46**
21	Complex words	3–6	3–6	3–6	3–6	3–**5**
22	Function words	3–6	3–6	3–6	3–6	3–6
23	Nonwords	3–6	3–6	3–6	**2**–**4**	**2**–**4**
Writing						
24	Copying	27	**9**	26	**25**	**25**
25	Naming	20	20	**12**	**9**	**12**
26	Dictation	28	**21**	**18**	**24**	**16**
Written description						
27	Content	19	**8**	**1**	13	11
	Form	6	**3**	**0**	**4**	**4**
	Total	25	**11**	**1**	17	15

N.T.: not tested. Bolded scores are below cutoff threshold and indicate impaired performance.

## Data Availability

The original data presented in the study are openly available in *CORA. Repositori de Dades de Recerca* at https://doi.org/10.34810/data2543.

## Supplementary Materials

The following supporting information can be downloaded at: https://www.mdpi.com/article/10.3390/brainsci15111193/s1, File S1: Complete transcriptions of oral descriptions.

## Abbreviations

The following abbreviations are used in this manuscript:

CAT	Comprehensive Aphasia Test
CAT-CAT	Comprehensive Aphasia Test (Catalan version)
GDS	Global Deterioration Scale
lvPPA	Logopenic variant of primary progressive aphasia
MMSE	Mini-Mental State Examination
nfvPPA	Nonfluent variant of primary progressive aphasia
PPA	Primary progressive aphasia
PWA	Participants with aphasia
PwPPA	Participants with primary progressive aphasia
svPPA	Semantic variant of primary progressive aphasia
TaM	Test de Alteración de la Memoria (Memory Alteration Test)

## Appendix A. Raw Scores and Error Data

This appendix presents the raw scores and data described and discussed in Section 3 and Section 4. It includes the severity scores for each subtest (Appendix A.1), along with types and frequencies of errors observed for each participant in different tasks (Appendix A.2).

### Appendix A.1. Severity Scores

The severity scores for each subtest and participant, calculated following the method described in Section 2.4 of the main text, are presented in Table A1.

**Table A1:** Severity scores on the CAT-CAT by participant.

Subtest		PPA1	PPA2	PPA3	PPA4
Cognitive screen					
1	Line bisection	0.00	0.00	N.T.	N.T.
2	Semantic memory	3.00	5.00	0.00	1.00
3	Word fluency				
	animals	1.28	1.34	1.14	1.10
	*p*	1.09	1.00	0.82	1.09
4	Recognition memory	2.00	4.00	0.00	2.00
5	Gesture object use	60.00	100.00	0.00	20.00
6	Arithmetic	2.00	2.00	2.00	2.00
Comprehension					
7	Spoken words	4.00	6.00	3.00	3.00
8	Written words	2.00	7.00	1.00	5.00
9	Spoken sentences	3.50	8.50	1.50	2.00
10	Written sentences	2.50	7.50	1.50	1.50
11	Paragraphs	2.00	4.00	1.00	1.00
Oral repetition					
12	Simple words	0.00	100.00	0.00	0.00
13	Complex words	0.00	13.00	0.00	0.00
14	Nonwords	0.00	3.00	0.00	0.00
15	Digit span	1.00	1.33	1.33	1.00
16	Word span	0.00	60.00	40.00	0.00
Oral naming					
17	Nouns	13.00	11.00	10.00	3.00
18	Verbs	0.00	40.00	20.00	20.00
Oral description					
19	Content	1.00	1.75	0.63	0.88
	Form	20.00	140.00	0.00	40.00
	Total	1.13	2.63	0.63	1.13
Reading					
20	Simple words	200.00	0.00	300.00	100.00
21	Complex words	0.00	0.00	0.00	0.00
22	Function words	0.00	0.00	0.00	0.00
23	Nonwords	0.00	0.00	20.00	20.00
Writing					
24	Copying	32.73	1.82	3.64	3.64
25	Naming	0.00	1.60	2.20	1.60
26	Dictation	2.69	3.85	1.54	4.62
Written description					
27	Content	1.10	1.70	0.60	0.60
	Form	3.00	6.00	2.00	2.00
	Total	1.27	2.09	0.73	0.91

N.T.: not tested.

### Appendix A.2. Error Types and Frequencies

The number and type of errors per participant in several subtests are presented in this section. For comprehension subtests, the error distribution is visualized in Figure A1 to better illustrate the pattern, while Table A2 summarizes the errors observed in the naming subtests.

**Table A2:** Number and type of errors per participant in the oral naming subtests from the CAT-CAT.

	Error Type	PPA1	PPA2	PPA3	PPA4	Total
Naming subtests						
	No response	5	7	10	0	22
	Semantic paraphasia	4	0	0	2	6
	Spanish	1	2	1	1	5
	Circumlocution	4	0	0	0	4
	Perseveration	0	2	0	0	2
	Phonetic paraphasia	0	0	0	1	1
Verbal fluency						
	Repetitions	1	0	0	1	2
	Spanish	0	0	2	2	4
	Circumlocutions	0	0	0	2	2
	Other categories	0	0	1	3	4

### Appendix A.3. Descriptions Tasks

The results for the oral and written description tasks are presented in Table A3, where scores falling below the cutoff threshold are bolded. The content variables include the number of correct relevant nouns, verbs, and content units produced to evaluate discourse efficiency; whereas the form variables, which assess grammaticality and grammatical complexity, and fluency in the oral description subtest, are evaluated using a Likert scale. In this case, the two researchers independently assigned identical scores. For further details, see Salmons et al. [19].

**Table A3:** Results on the description tasks from the CAT-CAT. Scores below the cutoff are in bold.

Subtest		Maximum	PPA1	PPA2	PPA3	PPA4
Oral description						
	Nouns	8	**4**	**4**	6	6
	Verbs	5	4	**1**	4	**2**
	Content units	6	3	**0**	4	4
	Content (total)	19	11	**5**	14	12
	Fluency	3	**2**	**0**	3	**1**
	Grammaticality	3	3	**1**	3	3
	Complexity	3	3	**1**	3	3
	Form (total)	9	**8**	**2**	9	**7**
Total		28	**19**	**7**	23	**19**
Written description						
	Nouns	8	4	**1**	7	5
	Verbs	5	**2**	**0**	3	3
	Content units	6	2	**0**	3	3
	Content (total)	19	**8**	**1**	13	11
	Grammaticality	3	2	**0**	2	2
	Complexity	3	**1**	**0**	2	2
	Form (total)	6	**3**	**0**	**4**	**4**
Total		25	**11**	**1**	17	15

In Table A4, the number of words in each category produced by the participants is presented. The word count excludes utterances that were immediate repetitions of what the participant or the speech-language pathologist had just said. We also analyzed the acoustic characteristics of the participants’ descriptions.

**Table A4:** Number of words produced by category and participant.

Category	PPA1	PPA2	PPA3	PPA4
Nouns	14	6	10	18
Verbs	20	7	17	20
Adjectives	1	0	0	0
Adverbs	13	6	9	2
Pronouns	9	4	12	6
Determiners	19	6	10	17
Conjunctions	14	1	11	5
Interjections	13	1	8	2
Fillers	2	3	3	2

In Table A5, the number and segment durations of each participant are presented.

**Table A5:** Summary of segment durations per participant.

Participant	Segment Type	N	Mean (SD)	Range (s)	Total Duration (s)
PPA1	Speech	37	1.52 (1.01)	0.40–5.52	56.32
	Silent pause	22	1.22 (1.23)	0.37–5.24	26.83
	Filled pause	1	1.29 (—)	1.29–1.29	1.29
PPA2	Speech	12	1.79 (1.11)	0.64–4.13	21.53
	Silent pause	8	6.20 (8.29)	0.71–22.13	49.59
	Filled pause	2	1.81 (0.48)	1.47–2.15	3.62
PPA3	Speech	16	2.03 (1.33)	0.30–4.82	32.40
	Silent pause	10	1.03 (0.76)	0.32–2.87	10.32
	Filled pause	4	1.42 (0.51)	0.75–1.99	5.69
PPA4	Speech	36	1.51 (0.83)	0.56–4.77	54.40
	Silent pause	29	2.58 (3.17)	0.34–16.73	74.89
	Filled pause	2	1.18 (0.45)	0.85–1.50	2.35

Values are reported as mean duration in seconds with standard deviation in parentheses, total cumulative duration, number of segments (N), and duration range (minimum–maximum).

## Appendix B. Sample Responses

This appendix contains samples of oral and written responses to illustrate the response patterns and errors discussed in the main text.

### Appendix B.1. Oral Responses

The transcriptions and approximate translations of the participants’ sample oral descriptions of the image in subtest 19 are provided. The duration of silent pauses is indicated within parentheses in seconds. Spanish words are in italics. The complete transcription of the oral descriptions can be consulted in the Supplementary Material.

1. ppa1: Aquí… (0.97) fa… (0.8) *pues* (0.5) *algo* de-de parlar (0.51) amb la dona aquesta. (0.96) No?
  *Translation*: Here… (the child) does… well something of-of talking with this woman. No?
2. ppa2: El gat… (2.01) Els-els llibres… (11.66) No ho sé.
  *Translation*: The cat… The-the books… I don’t know.
3. ppa3: Una mà que s’ha quedat dormint… (0.9) I el nen sembla que està tu… (0.31) q-que està… como que le vol gridar, perquè se, t- (0.75, filled) (1.02) aah, *porque* es treuen xxx, mira, los llibres. (0.73) I el gat. (2.87) Sí, *por eso, por eso*, el, el, el gat, perquè la iai-, la mama no se caigui. […]
  *Translation:* A hand (paraphasia for ‘mother’) that has fallen asleep… And the boy seems to be… that he is… as if he wants to shout at her, because he, t- aaah, because they take out (unintelligible), look, the books. And the cat. Yes, that’s why, that’s why, the, the, the cat, so that the granny, the mother doesn’t fall.
4. ppa4: El nano. (2.09) El nano (1.37) jugant amb una, amb una… (16.73) El nano jugant amb una, amb unaa… (1.02) amb unaa… eeh (1.5, filled) nina. (1.63) Amb una nina.
  *Translation*: The kid. The kid… playing with a, with a… The kid playing with a, with a… with a… uh… doll. With a doll.

### Appendix B.2. Written Responses

Sample written responses from participants are reproduced below. Examples were included with permission. Clinician annotations have been digitally removed for presentation clarity.
