# Supplementary material for: Exploring Language Impairment in Catalan-Dominant Bilinguals with Primary Progressive Aphasia: Preliminary Data
Source: Brain Sci. 2025 Nov 4;15(11):1193. doi: 10.3390/brainsci15111193 (PMC12650411; doi:10.3390/brainsci15111193)
Supplement: Supplementary file 1 [file brainsci-15-01193-s001.zip › brainsci-3923615-supplementary/supplementary/PPA_CATCAT_supplementary material.pdf]

Supplementary Materials: Exploring language impairment in Catalan-dominant bilinguals with primary progressive aphasia: preliminary data

Io Salmons[1]* and Helena Muntané-Sánchez[2]

1. Introduction

This file contains transcriptions and approximate translations of the oral descriptions of the image in subtest 19 of the Catalan version of the *Comprehensive Aphasia Test* [1,2] produced by four patients with primary progressive aphasia (PPA). In the transcriptions, the examiner is annotated as SLP, and the codes PPA1, PPA2, PPA3, and PPA4 refer to the four participants with PPA. In addition, silent and filled pauses are indicated in seconds within parentheses, whereas unintelligible speech is marked with XXX. A dash (–) indicates that a word was cut off by the speaker, either self- or other-initiated. Non-verbal actions such as pointing are annotated in double parentheses (( )). Words in Spanish are written in italics.

2. Transcriptions of oral descriptions

2.1. PPA1

PPA1: Aquí veig un gat. Aquí, uns llibres. Aquí, un d'això de-de *algo*, de aigua, amb alguna–

SLP: Molt bé. Mira, et trec el llapis perquè si no, es pinta una miqueta, *vale*? Aquí, una peixera.

PPA1: Una peixera. Exacte. (1.41) Aquestes no les veu, eh.

SLP: *Bueno*, això està al fons, no et preocupis. Només *lo* important, que està passant aquí?

PPA1: Sí. (4.1) És aquí ((points to the loudspeakers)), *bueno* aquí és com si fos el (0.51) el d'allò d'un cotxe. (1.05) I-i va…

SLP: El què d'un cotxe?

PPA1: Vas en un cotxe (0.57) i, i, i… (0.91) i té, és que, clar…

SLP: Ah, com els *faros* una miqueta?

PPA1: Sí.

SLP: *Vale*, fixa't en la imatge general. Què hi passa aquí?

PPA1: (1.65) Pues aquí està dormint. (0.94)

SLP: Exacte.

PPA1: Bueno, dormint. (1.28) Eeh... (1.29, filled) Una senyora que està (0.77) aquí amb uns… (1.07) llibres o…

SLP: Perfecte.

PPA1: XXX (0.4) I-i i aquí, (0.44) i aquí *pues* hi ha un petit (0.37) noi o noia, (1.76) al terra.

SLP: I què fa?

PPA1: (1.58) Aquí... (0.97) fa... (0.8) *pues* (0.5) *algo* de-de parlar (0.51) amb la dona aquesta. (0.96) No?

SLP: Molt bé. Exacte. I aquí, què em deies? El gat què està fent?

PPA1: (2.05) El gat estava veient (0.63) els d'allòs que hi han aquí dins de la… (5.24) dins de la… pf, *cómo* se diu?

SLP: Dins de la peixera.

PPA1: De la peixera.

SLP: Exacte. Molt bé. I què passarà?
PPA1: (0.71) Pf, *pues* que cauran tots aquests llibres.
SLP: Exacte. I per què cauen?
PPA1: No?
SLP: Exacte. I per què cauen?
PPA1: Cauen perquè ha saltat el gat.
SLP: Exacte. Perquè ha estat el gat qui els ha tirat.
PPA1: Ha estat el gat qui els ha tirat.

English translation:

PPA1: Here I see a cat. Here, some books. Here, one of those of-of something, of water, with some–
SLP: Very good. Look, I'll take the pencil away because otherwise, it will draw a little, okay? Here, a fish tank.
PPA1: A fish tank. Exactly. These she doesn't see, eh.
SLP: Well, that's in the background, don't worry. Just focus on what's important. What's happening here?
PPA1: Yes. It's here ((points to the loudspeakers)), well here it's as if it were the... that thing from a car. And-and goes. . .
SLP: The what from a car?
PPA1: You go in a car and, and, and. . . and it has, it's that, of course. . .
SLP: Ah, like the headlights a little?
PPA1: Yes.
SLP: Okay, look at the general image. What is happening here?
PPA1: Well, here is sleeping.
SLP: Exactly.
PPA1: Well, sleeping. Eeh... A lady who is here with some. . . books or. . .
SLP: Perfect.
PPA1: (unintelligible) And-and here, and here well there is a little boy or girl, on the floor.
SLP: And what is he doing?
PPA1: Here... does... well something of-of talking with this woman. No?
SLP: Very good. Exactly. And here, what were you telling me? The cat what is it doing?
PPA1: The cat was looking at the things that are here inside the. . . inside the. . . pf, how do you call it?
SLP: Inside the fish tank.
PPA1: Of the fish tank.
SLP: Exactly. Very good. And what will happen?
PPA1: Pf, well that all these books will fall.
SLP: Exactly. And why do they fall?
PPA1: No?
SLP: Exactly. And why do they fall?
PPA1: They fall because the cat jumped.
SLP: Exactly. Because it was the cat who knocked them over.
PPA1: It was the cat who knocked them over.

2.2. PPA2

PPA2: Aquests... (1.47, filled) fot e-el nas... (21.87) Aquí ja no, no està. (16.61)
SLP: I aquí? ((pointing to the woman))

PPA2: (6.51) Aquí està la... la senyora que jo.. no sé... (4.18) No sé. (0.97) El nen es-es-està... (0.71) eh, en fin, eh-eh, es... (1.47,filled) (2.12) Està. Es-es-e-el nen està, eh, est, està cuidant. (1.05) està cuidant, no ho sé. (0.65)
SLP: *Vale*. Està assenyalant, no?
PPA2: L'escala, l'escala.
SLP: I per aquí?
PPA2: (0.92) El gat... (2.01) Els-els llibres... (11.66) No ho sé.

English translation:

PPA2: These... eh, eh, um, eh... poke a-a-around... Here no longer, not there.
SLP: And here?
PPA2: Here is the... the lady that I... don't know... I don't know. The boy i-i-is... eh, anyway, eh-eh, he... is. I-it-the boy is, eh, is, is taking care of, is taking care of, I don't know.
SLP: Okay. He's pointing, right?
PPA2: The stairs, the stairs.
SLP: And around here?
PPA2: The cat... The books... I don't know.

2.3. PPA3

PPA3: Una mà que s'ha quedat dormint... (0.9) I el nen sembla que està tu… (0.31) q-que està… como que le vol gridar, perquè se, t- (0.75, filled) (1.02) aah, *porque* es treuen XXX, mira, los llibres. (0.73) I el gat. (2.87) Sí, *por eso, por eso*, el, el, el gat, perquè la iai-, la mama no se caigui. (0.91) Però… (1.7) Y este… (0.6) eh, laa… eeh… (1.75, filled) Eso de… mm, sí, de, de (1.5, filled) *pescaditos que hay aquí, esas cosas, cómo se llaman, no sé*. (0.35) *Que hay ahí*… sí. (0.99) *Y, y, y se quieren meter aquí a cogerse los* XXX *los paja–*. (1.12)
SLP: Molt bé. Ens deixem *algo*?
PPA3: (0.77) Eh?
SLP: Hi ha *algo* més que sigui important.
PPA3: (1.26) *Bueno*, *pues* poder, poder *pues*… (6.68)
SLP: O ja està, també està bé.
PPA3: *Bueno*, les flors aquestes. No sé t– (1.45, filled). Per això s'ha sortit a fora.

English translation:

PPA3: A (unintelligible) that has fallen asleep... And the boy seems to be… that he is… as if he wants to shout at her, because he, t- aaah, because they take out (unintelligible), look, the books. And the cat. Yes, that's why, that's why, the, the, the cat, so that the granny, the mother doesn't fall. But… And this… eh, the… eeh… That of… mm, yes, of, of little fishes that are here, those things, how do they call them, I don't know. What's in there… yes. And, and, and they want to get in here to grab the (unintelligible) the (unintelligible).
SLP: Very good. Are we missing something?
PPA3: Eh?
SLP: Is there something else that is important?
PPA3: Well, so maybe, maybe so…
SLP: Or that's it, that's also fine.
PPA3: Well, these flowers. I don't know t–. That's why it went outside.

2.4. PPA4

PPA4: Què hi veig?
SLP: Sí, què hi passa?
PPA4: Hi veig (0.65) una tauleta (1.54) amb llibres, (1.24) una estanteria amb llibres i un gat. (3.02) Una dona dormint. (2.51) Fent, fent la migdiada. (1.64) Un-un, uun planta-a... un, una planta. (3.56) Una planta. (4.02) És massa liàs, eh. (2.27) Haig descriure tot?
SLP: M'has d'explicar tot el que passa aquí a la imatge.
PPA4: (8.41) *Bueno.* (2.43) Haig-ee-el gat (1.22) espanta els llibres (1.0) i cuida (1.39) dee... (1.42) de nooo... (8.74) És quee... em sembla... eh, eh, (0.86, filled) una tonteria.
SLP: Per què?
PPA4: (2.66) Perquè... (1.82) relacionar (0.35) tot el que hi ha... (1.99) Una dona dormint, (0.42) fent la migdiada.
SLP: I aquí?
PPA4: (5.06) El nano. (2.09) El nano (1.37) jugant amb una, amb una... (16.73) El nano jugant amb una, amb unaa... (1.02) amb unaa... eeh (1.5, filled) nina. (1.63) Amb una nina. (1.76) I la dona darmint, (0.67) dormint. (4.42) Els llibres... (2.47) un tirar... tirar cordes. (1.52) Tirar cordes.

English translation:

PPA4: What do I see?
SLP: Yes, what is happening?
PPA4: I see a small table with books, a bookshelf with books and a cat. A woman sleeping. Taking, taking a nap. A-a, a-a plant... a, a plant. A plant. It's too messy. Do I have to describe everything?
SLP: You have to explain everything that is happening in the image.
PPA4: Well. The cat scares the books and takes care of... of not... It's that... it seems to me... eh, eh, nonsense.
SLP: Why?
PPA4: Because... to link everything that is here... A woman sleeping, taking a nap.
SLP: And here?
PPA4: The boy. The boy playing with a, with a... The boy playing with a, with a... with a... eeh doll. With a doll. And the woman sleeping, sleeping. The books... a... throwing... throwing ropes. Throwing ropes.

References

1. Swinburn, K.; Porter, G.; Howard, D. *Comprehensive Aphasia Test*; Psychology Press: Hove, 2005.
2. Salmons, I.; Rofes, A.; Gavarró, A. *Prova integral d'afàsia. Llibre d'ítems*; Servei de Publicacions de la UAB: Bellaterra, 2021.

Disclaimer/Publisher's Note: The statements, opinions and data contained in all publications are solely those of the individual author(s) and contributor(s) and not of MDPI and/or the editor(s). MDPI and/or the editor(s) disclaim responsibility for any injury to people or property resulting from any ideas, methods, instructions or products referred to in the content.
